# Supplementary figures and images for: Comprehensive Study of Germline Mutations and Double-Hit Events in Esophageal Squamous Cell Cancer
Source: Front Oncol. 2021 Apr 6;11:637431. doi: 10.3389/fonc.2021.637431 (PMC8056176; doi:10.3389/fonc.2021.637431)

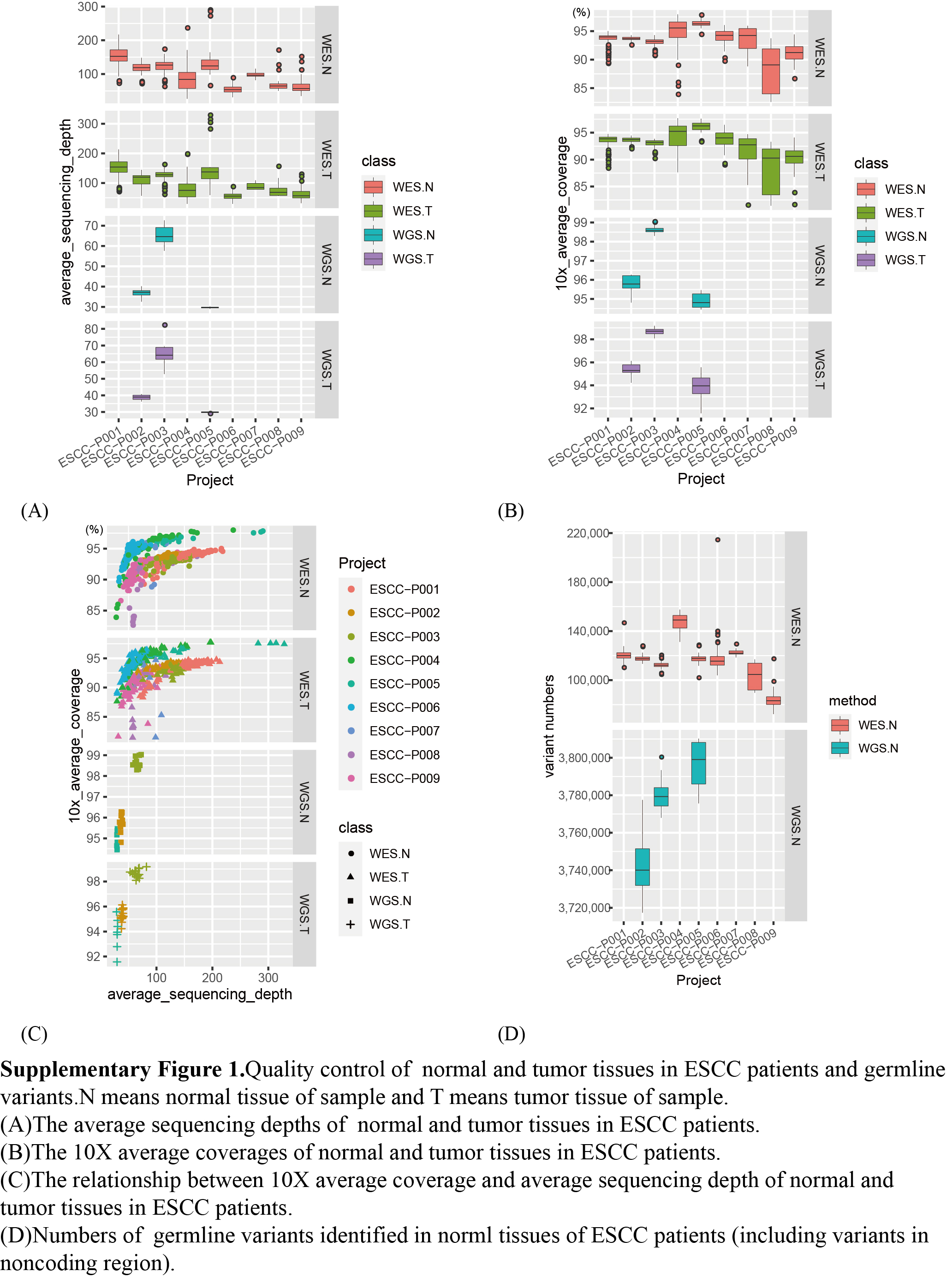

Supplement: Supplementary file 13 [file Image_1.JPEG]

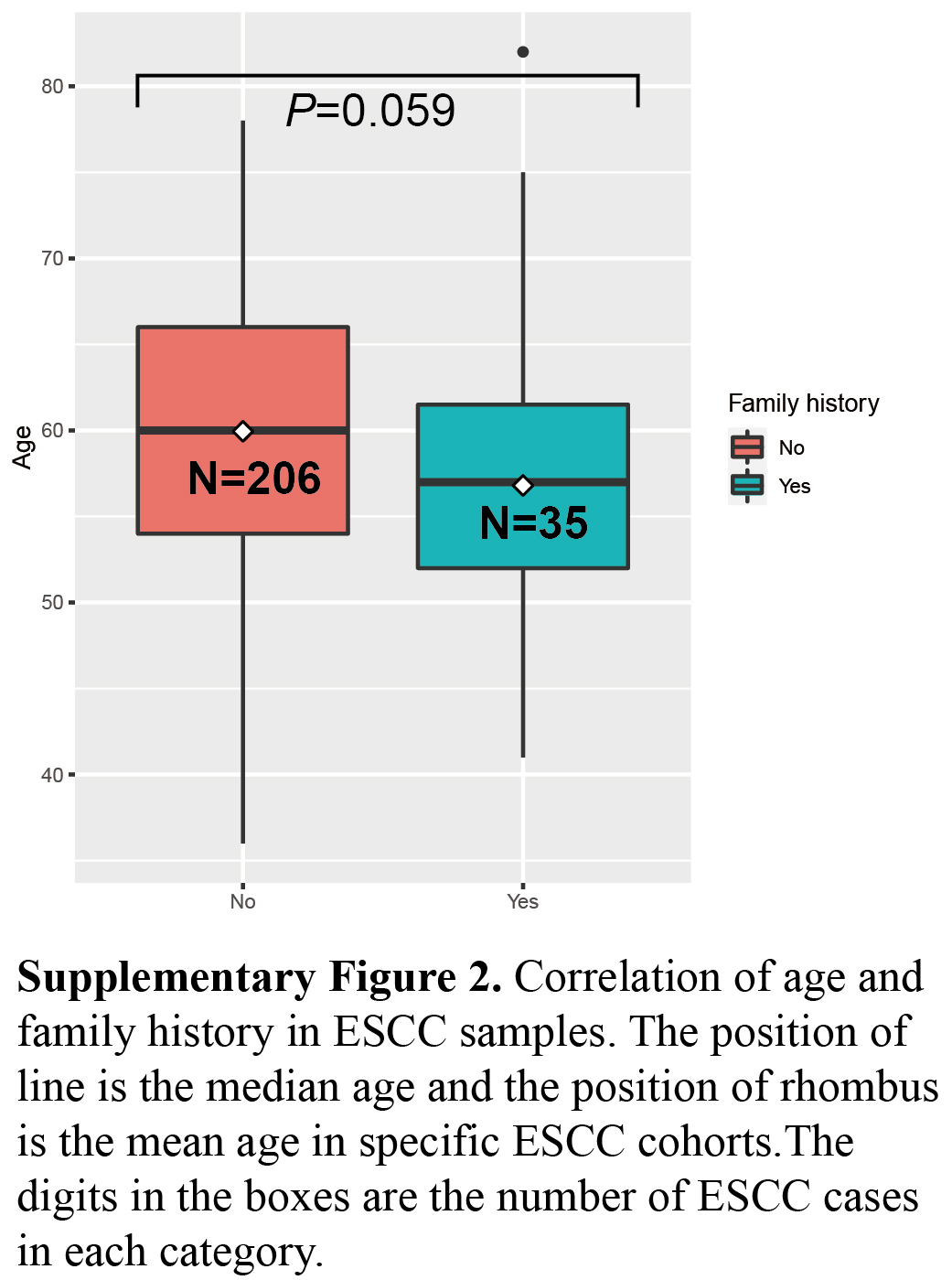

Supplement: Supplementary file 14 [file Image_2.jpg]

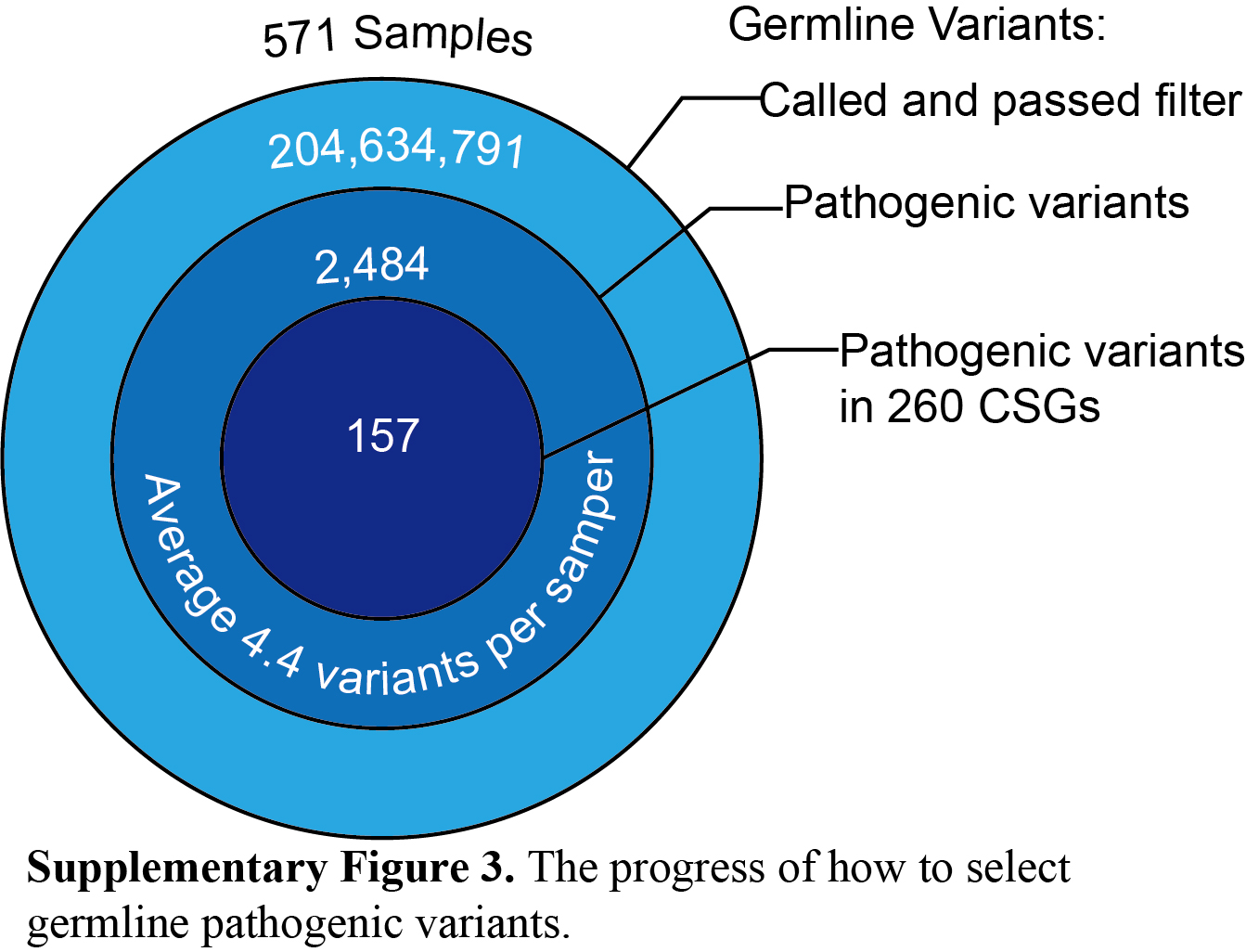

Supplement: Supplementary file 15 [file Image_3.JPEG]

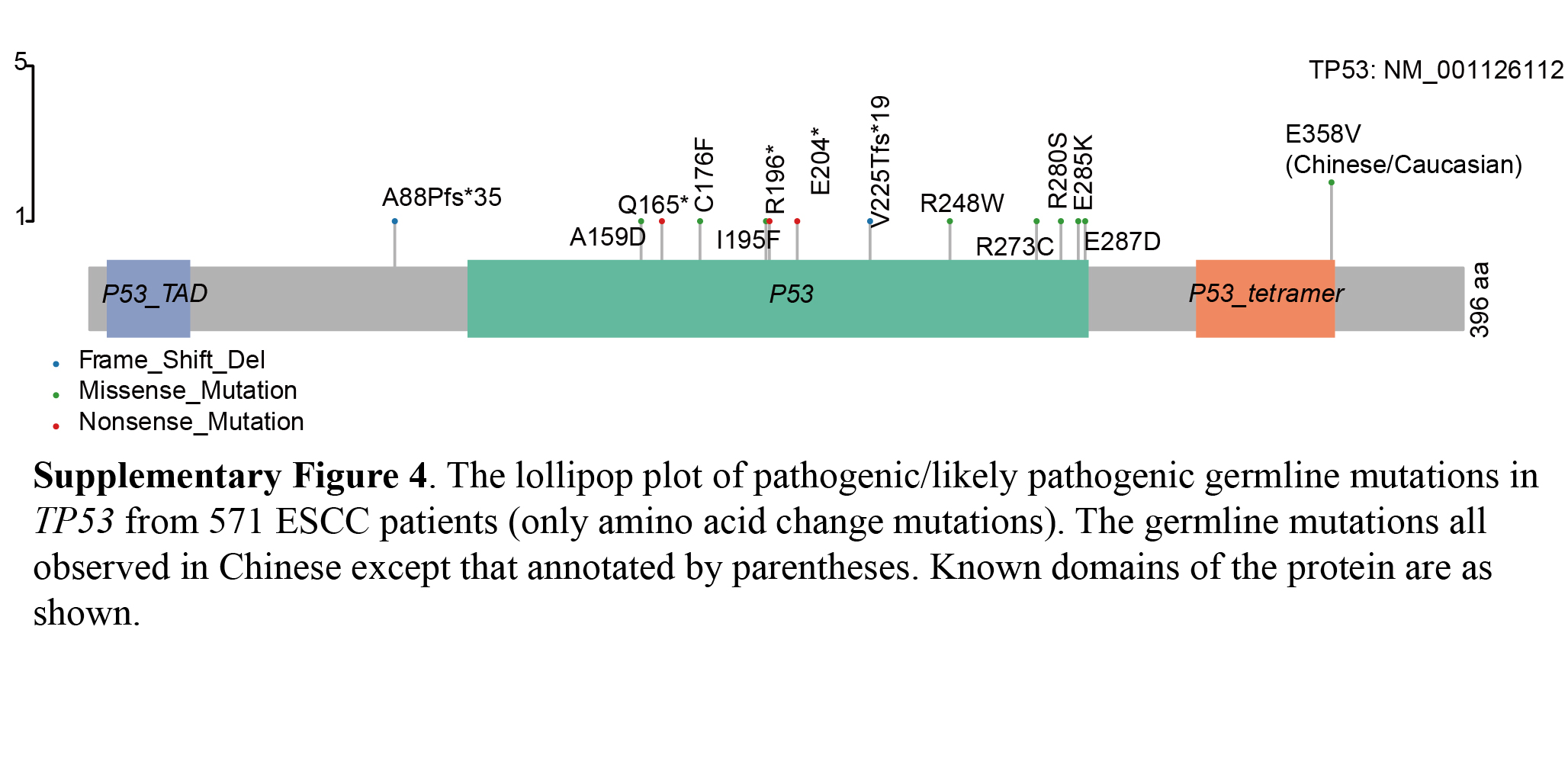

Supplement: Supplementary file 16 [file Image_4.jpg]

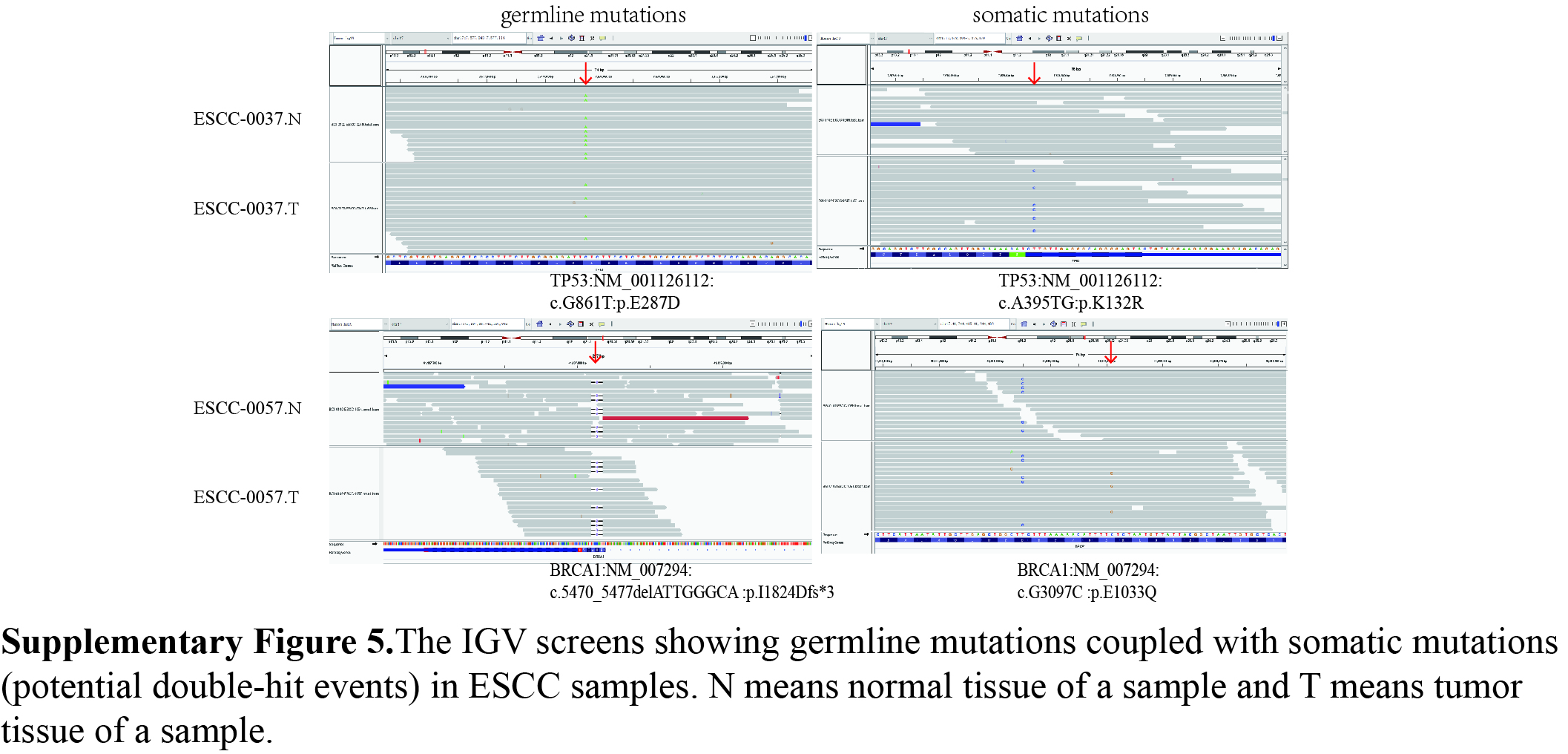

Supplement: Supplementary file 17 [file Image_5.JPEG]

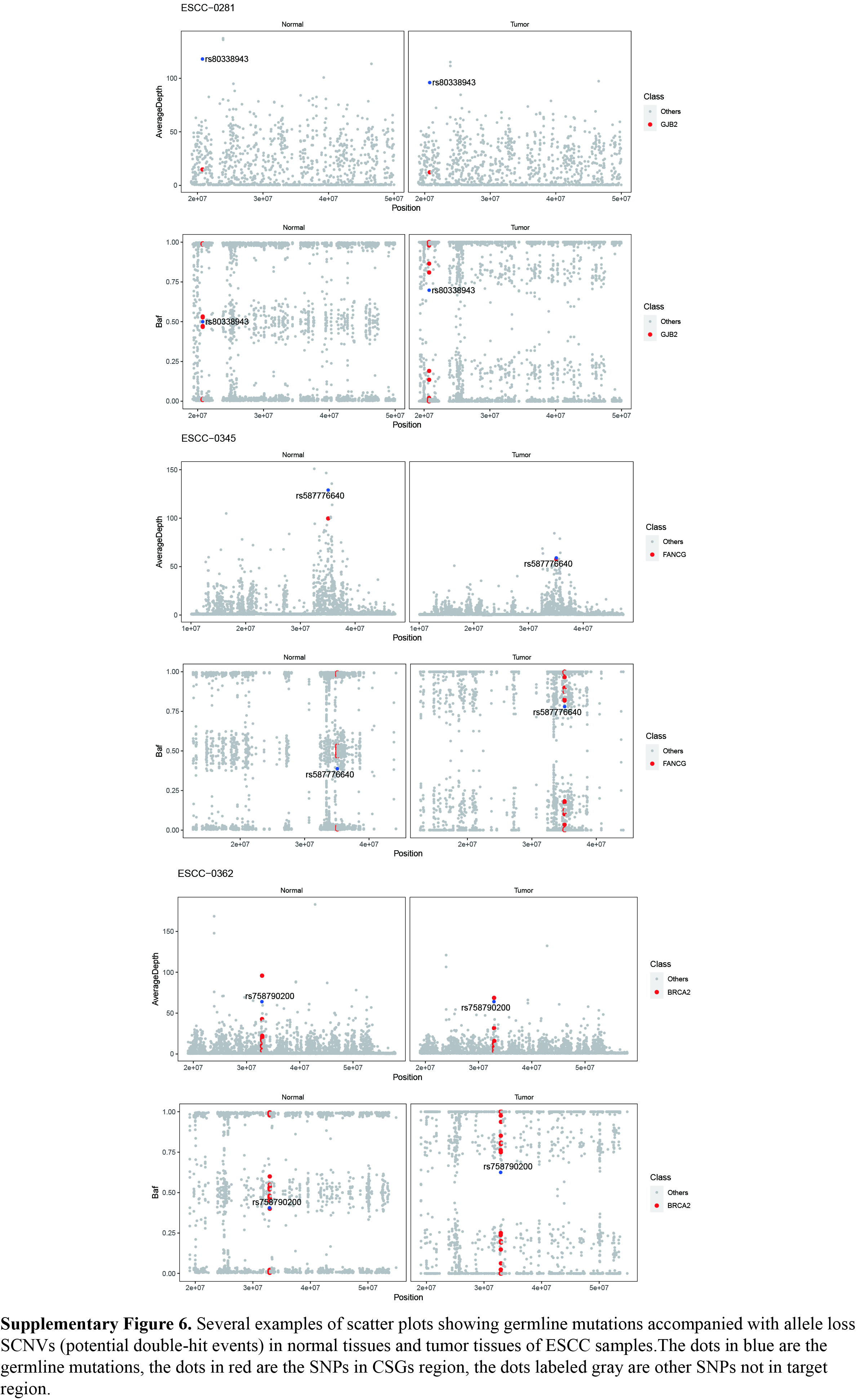

Supplement: Supplementary file 18 [file Image_6.JPEG]

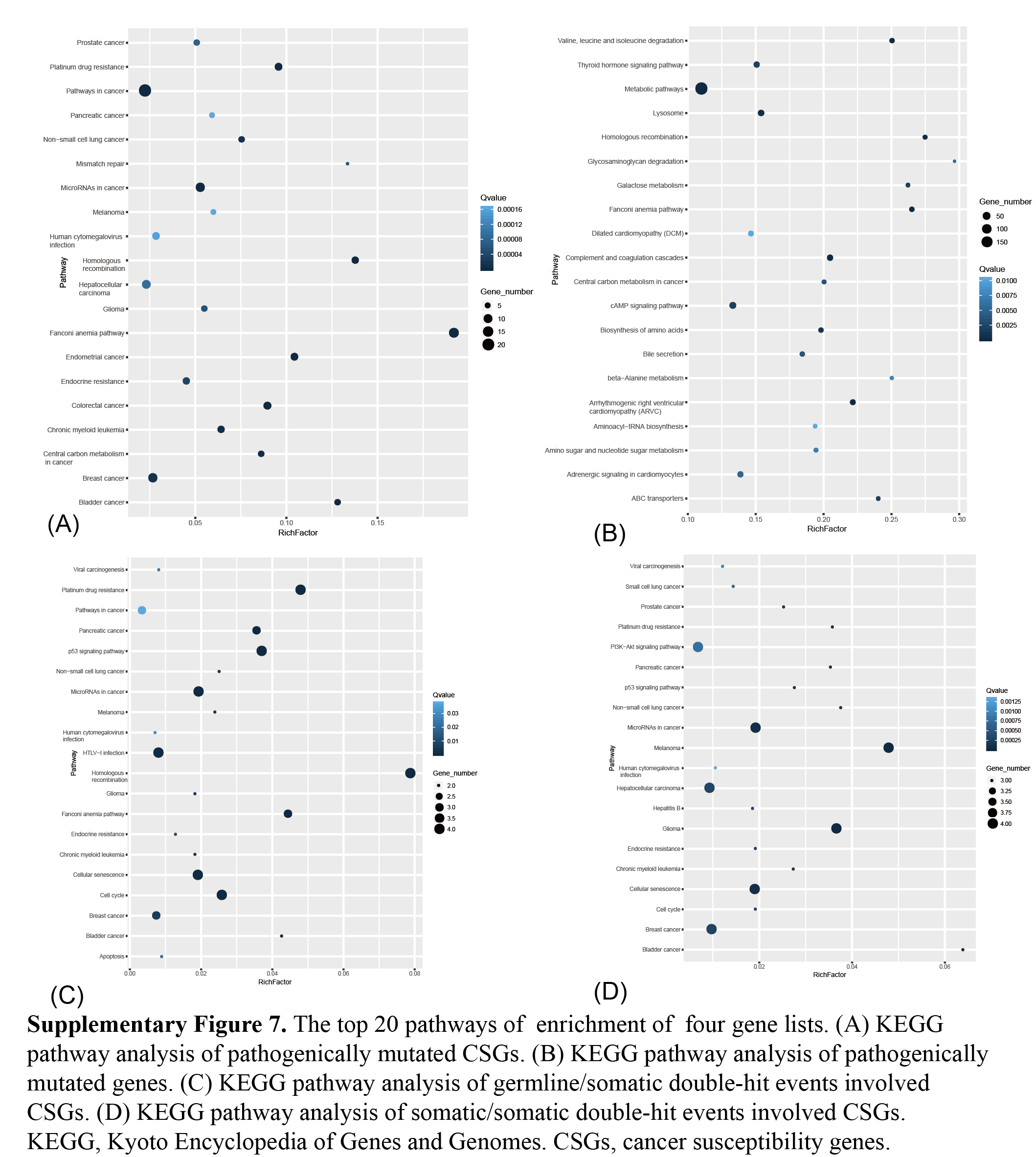

Supplement: Supplementary file 19 [file Image_7.jpg]

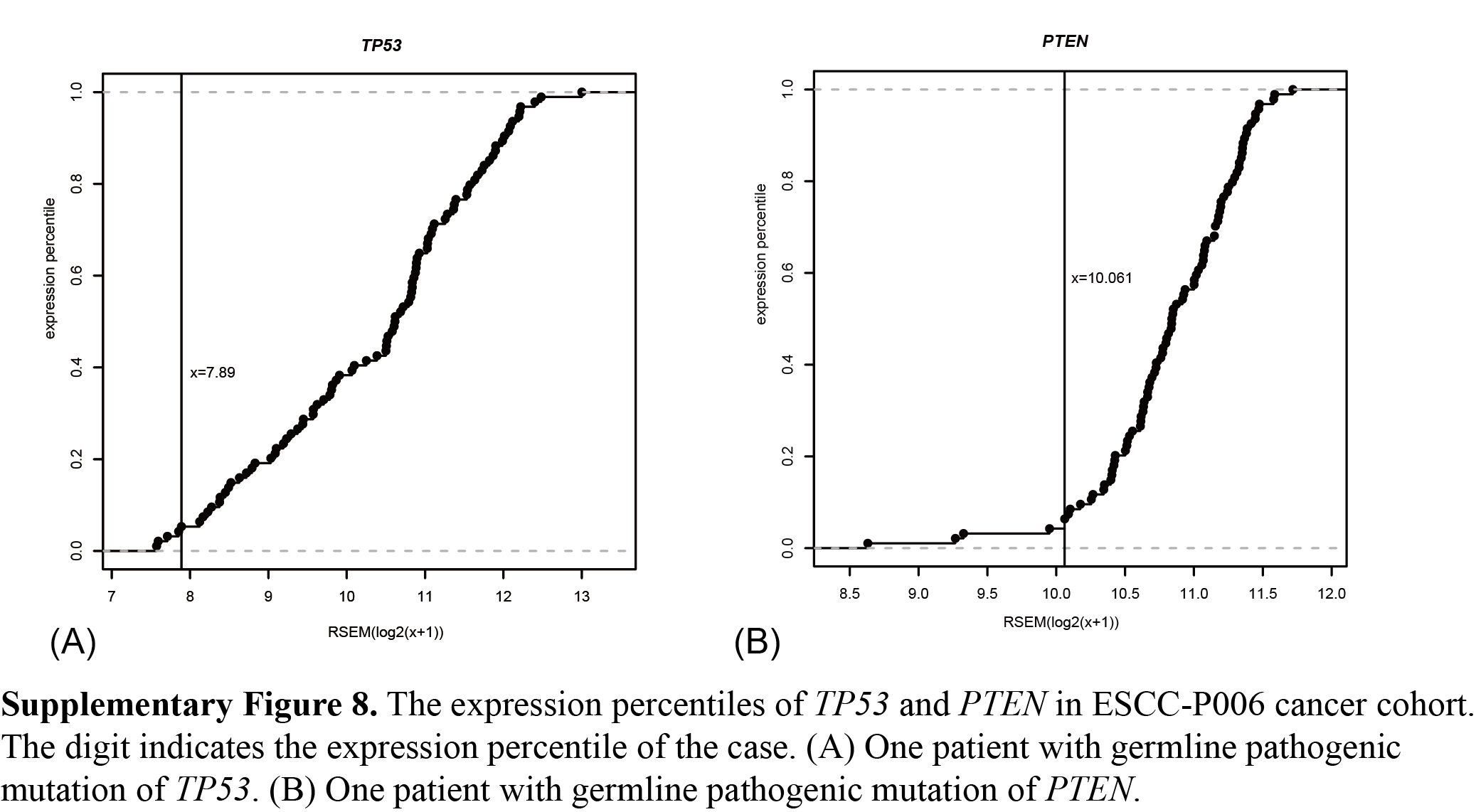

Supplement: Supplementary file 20 [file Image_8.JPEG]
